# Immediate restoration of fixed full-arch prostheses placed on implants in both fresh and healed sockets using the flat one-bridge technique: a 7-year retrospective study

**DOI:** 10.1186/s12903-021-01988-0

**Published:** 2021-12-03

**Authors:** Simone Marconcini, Enrica Giammarinaro, Ugo Covani, Andrea Mascolo, Guerino Caso, Marco Del Corso

**Affiliations:** 1 Tuscan Dental Institute, Foundation for Dental Clinic, Research and Continuing Education, Versilia General Hospital, Lido Di Camaiore, LU Italy; 2 Fernando Pessoa University, Porto, Portugal; 3 Naples, Italy; 4 Turin, Italy

**Keywords:** Postextraction sockets, Immediate implants, Immediate loading, Immediate restoration, Flat abutment

## Abstract

**Background:**

The aim of this retrospective study was to document the long-term clinical efficacy of a surgical-prosthetic technique (the flat one-bridge technique) involving the immediate restoration of both postextraction and nonpostextraction implants supporting full-arch restorations.

**Methods:**

Implants were placed by adapting the axis to the available bone. Flat definitive abutments were connected during surgery and never disconnected to compensate for eventual implant disparallelism. Bone grafting was performed when needed. The patients received a screw-retained provisional restoration within 48 h of surgery and a final screw-retained prosthesis within 1 year.

**Results:**

Sixty-six patients received 494 implants distributed in 75 prostheses. The median follow-up was 86 months (range 82–168 months). Only three implants had failed at the last follow-up. Implant survival was 99.6%.

**Conclusion:**

The flat one-bridge prosthetic protocol is a viable procedure with excellent long-term outcomes. No difference in clinical success could be observed between postextractive and nonpostextractive implants.

## Background

The establishment of new, predictable techniques for treating edentulous patients or patients with failing dentition using immediate fixed full-arch restorations on dental implants has dramatically improved the standard of care for patients, often allowing fast, cost-effective rehabilitation of the jaws [1–3].

Although the traditional Branemark approach was meant to be a two-step treatment in which implants are left unloaded to heal for at least 3 to 6 months [4], several upgrades of the procedure have been introduced that have led to the advent of immediate loading at postextraction sites, allowing fixed full-arch restoration of the jaws with no waiting time [5, 6]. An immediate loading protocol allows patients to wear their implant-supported prostheses within the first week after implant surgery and avoid secondary surgery and provisional prostheses, which are often barely functional [7].

Patients’ quality of life is severely affected by tooth loss, and implant-retained prostheses have been documented as a much more satisfying solution than removable dentures for patients with failing dentition [8]. A recent analysis suggested that there were no significant differences between immediate-loading and delayed-loading full-arch protocols in terms of clinical, radiological, or patient-related outcomes [9], meaning that shortened procedures should always be adopted when medical indications and oral surgeon experience is equal. In fact, regardless of how appealing it may sound, immediate implant placement (IIP) with immediate loading carries several feasibility concerns. Concerns about local infection, achieving primary stability of the implant, and respecting the intended implant position are only a few of the short-term surgical tasks that directly influence the outcome of IIP. Compliance with those requirements depends primarily on the clinician’s expertise, the instruments used and the implant design [10].

High survival rates have been reported for full-arch prostheses placed on implants in both healed and extraction sites, with no difference in implant failure rates between the two conditions [11, 12]. However, most studies concerning full-arch restorations on failing dentition have addressed the efficacy of computer-planned guided surgery and often fail to mention cases in which implants were placed without drilling templates [13]. It must be stressed that the survival rates of immediate implants depend on several factors that are far more important than the availability of a surgical guide: primary stability, implant design and dimension, surgical technique, and number of implants.

The aim of the present study was to evaluate the clinical outcome of implants placed in healed and extraction sites and immediately loaded with full-arch restorations by means of the flat one-bridge technique, which should allow an easy prosthetic phase and convenient passivation of the suprastructure [14, 15].

## Methods

The present retrospective cohort study analyzed data retrieved from patients treated at different clinics (main center: Istituto Stomatologico Toscana, Forte dei Marmi, LU, Italy) with fixed full-arch rehabilitations placed on dental implants between 2007 and 2019. The investigation was performed according to the principles embodied in the Helsinki Declaration of 1964, amended in 2008, for biomedical research involving human subjects. Due to the retrospective nature of the study, the need for ethical approval was waived by the UniCamillus Ethical Committee. The patients gave their written consent for anonymous data collection and analysis and scientific publication.

**Fig. 1:**
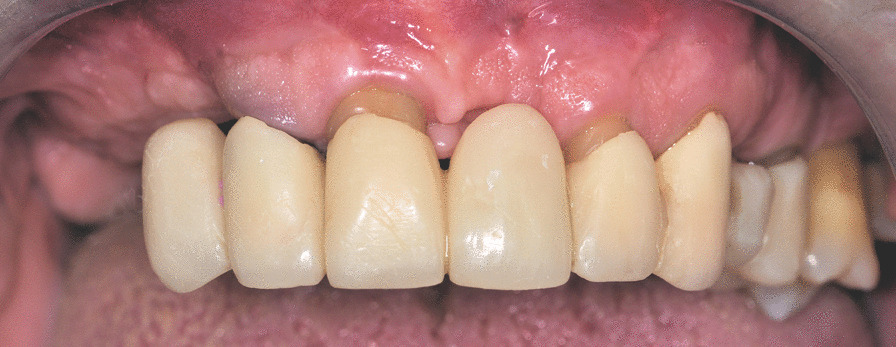
Intraoral photograph of a male patient with failing upper dentition

**Fig. 2:**
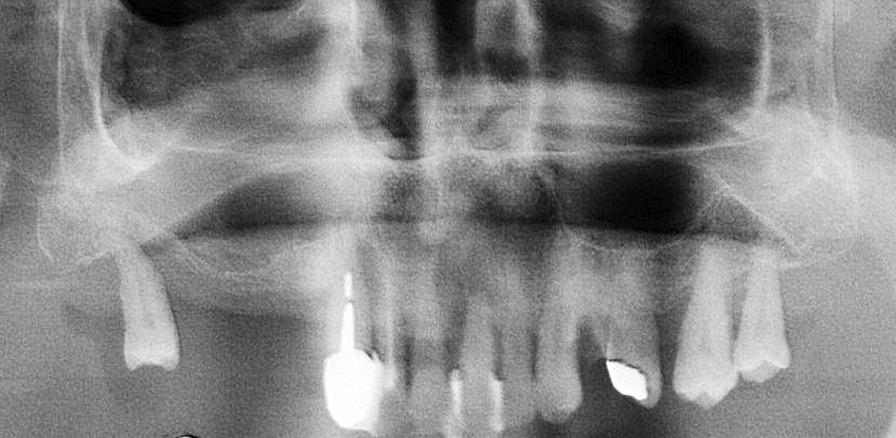
Panoramic radiograph of the same patient

Patients who had previously undergone extraction of their remaining failing teeth and immediate fixed full-arch rehabilitation were considered eligible for inclusion in the present study (Figs. 1, 2). Patients were included in the analysis if they met the following criteria: age ≥ 18 years, good systemic health, and compliance with adequate hygiene maintenance and follow-up visits. All patients received six or more implants positioned across the entire arch and stabilized with an insertion torque > 35 Ncm. The exclusion criteria were the presence of any local or systemic factor that might have contraindicated oral surgery, poor oral hygiene, heavy smoking (> 10/day), pregnancy, or a history of drug or alcohol abuse.

Data were collected regarding patient-related information (sex, age at surgery, systemic health status, smoking habits, oral hygiene), implant-related information (diameter, length, healed/extraction site, position, number of implants per arch, tilt, loading), and prosthetic rehabilitation (material, complication, follow-up). Any complications that occurred during or after the surgery were taken into account and summarized to determine the cumulative success and survival rate of both the implants and the entire rehabilitation process.

### Surgical procedure

Three experienced clinicians performed the surgeries according to standard protocols. The surgical procedure was performed under local anesthesia (articaine chlorhydrate 1:1,000,000 adrenaline, Septodont, Pennsylvania, USA), and all of the patients received 2 gr of amoxicillin 1 h before surgery and twice a day for 6 days thereafter (GlaxoSmithKline spa, UK). Each patient’s mouth was rinsed with ozonized water for 1 min before and after the intervention (Aquolab srl, Italy). Before implant placement, failing teeth were extracted using a minimally invasive technique, eventually with the aid of a magnetostrictive device (Magnetic Mallet, Osseotouch, Italy), with the intent of preserving the maximum amount of residual bone. Implants were placed in both the healed and postextraction sites depending on the specific situation; specifically, they were placed wherever primary stability could be achieved. The surgeons prepared the implant sites with standard drills of increasing diameter, with the final implant shoulder position at a bone level or slightly subcrestal. To ensure primary stability, the drilling protocol sometimes included underpreparation of the implant bed depending on the local bone density. The longest possible implants were placed in the postextraction sites, with the aim of achieving apical primary stability. Implants were inserted with a torque controller (Osstell, USA) to avoid excessive detrimental torque. All patients were treated with FOB over at least 6 Ossean surface implants (IntraLock, Boca-Raton, FL, USA) positioned across the entire arch and stabilized with an insertion torque of at least 35 Ncm. Ossean implants have an improved nanorough, low-calcium impregnated surface and the conic macrogeometry facilitates the achievement of primary stability and provides early osseointegration in the first weeks after surgery [16–18].

**Fig. 3:**
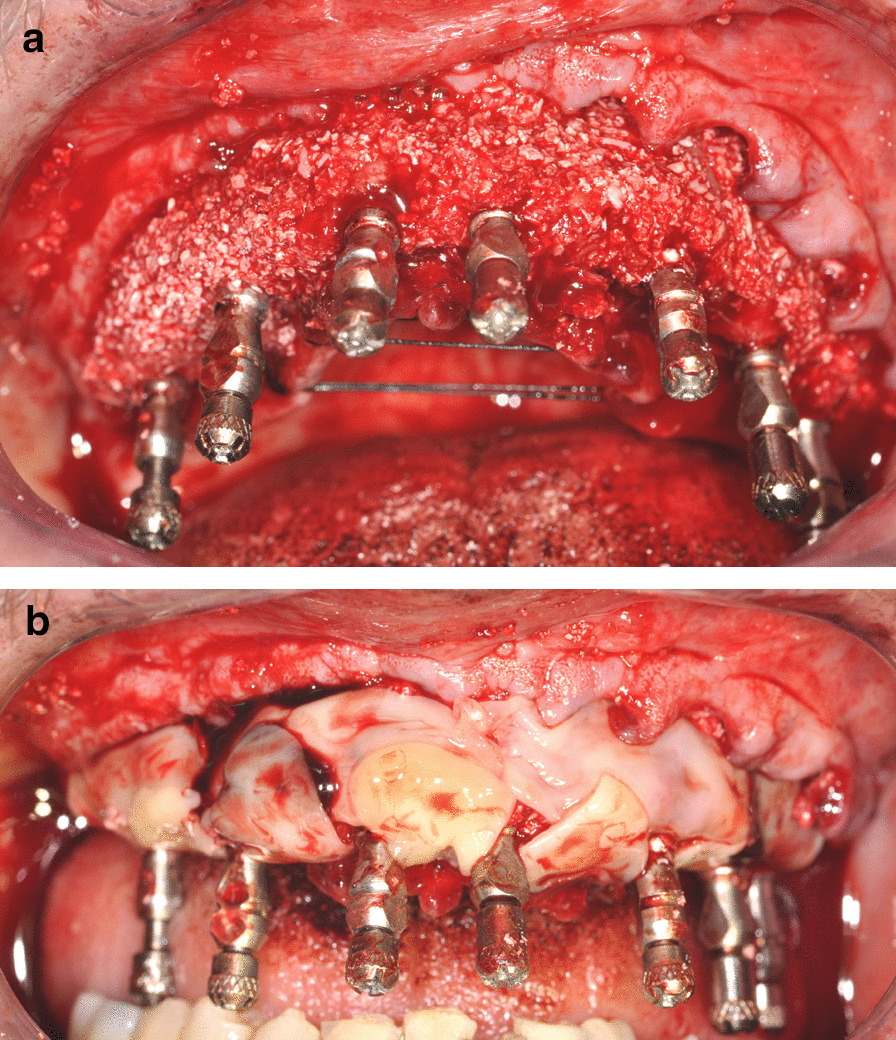
**a** Intraoral photograph of the upper arch immediately after surgery, with bone graft showing. **b** Intraoral photograph of the lower arch immediately after surgery showing L-PRF

If indicated [19], interventions were associated with bone augmentation procedures that combined corticospongious porcine grafts (GenOs, Roen, Turin, Italy) with leukocyte- and platelet-rich fibrin (L-PRF, Intra-Lock, Boca Raton, US) to enhance the biological effects of the biomaterial with autologous GF derived from a specific hemocomponent. Regeneration was mainly meant as a means of achieving pleasant soft tissue outcomes (Fig. 3a, b); thus, the use of biomaterials was limited to cases of severe hard and soft tissue atrophy with fair odds of achieving proper vascularization of the graft. Otherwise, a flapless approach was used whenever possible.

### Prosthetic procedure

**Fig. 4:**
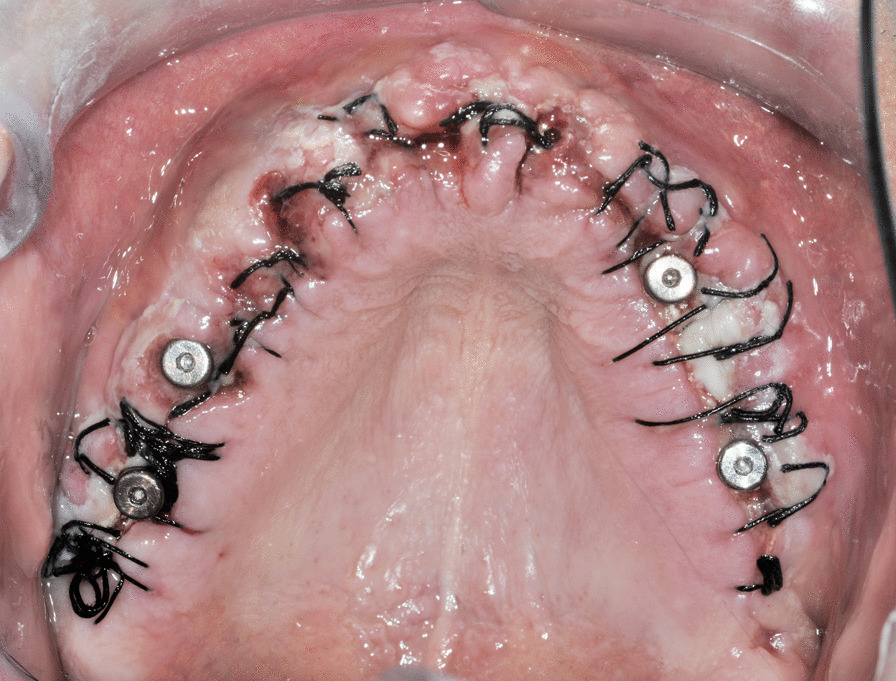
Intraoral photograph of soft tissue healing 72 h after surgery

Upon the achievement of sufficient primary stability (torque > 35 Ncm), the clinician connected the definitive abutments to the fixtures in order to screw in and adapt a fixed full-arch bridge (flat one-bridge (FOB)) within 72 h of the surgical procedure (Fig. 4). Flat abutments used were (flat abutment, IntraLock, Boca Raton, FL, US). Because they are connected to each implant, flat abutments can compensate for problems with the implant axis prior to the preparation of the implant-supported prosthesis, thereby facilitating the passive fit of the prosthetic framework over a flat pillar. These abutments were designed to reduce the forces that counteract a passive implant-to-casting fit, with the aim of decreasing the risk of biomechanical complications and helping to dissipate the forces acting on each single implant during cyclic loads, even in cases of disparallelism among the fixtures.

Provisional prosthetic rehabilitation included a fixed bridge screwed onto the flat abutments. The provisional frameworks were mostly fabricated with Cr-Co (72 arches) and covered with high-pressure resin or composite. Only 3 patients received provisional rehabilitation with metal-free glass-fiber frameworks covered with composite; 1 patient received a provisional braze-welded framework that was maintained definitively and covered with composite esthetic material.

**Fig. 5:**
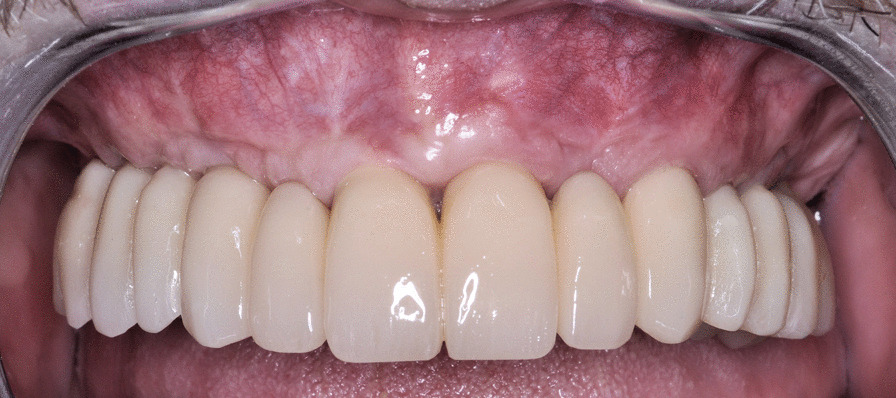
Intraoral photograph of soft tissue at a 4-month follow-up visit

**Fig. 6:**
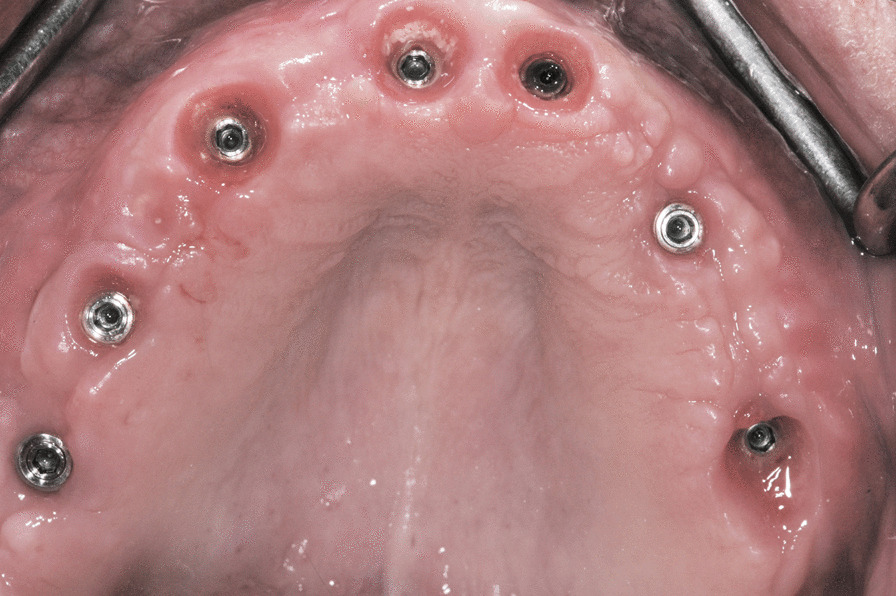
Intraoral photograph of the restoration at a 4-month follow-up visit

Generally, after a minimum of 4 months (Fig. 5), a definitive framework of passivated Cr-Co supporting esthetic composite teeth was fabricated (Fig. 6); one patient received a provisional braze-welded bar that was maintained definitively with composite occlusal coverage; twelve patients’ frameworks were replaced with a metal framework supporting ceramic teeth; and one patient’s framework was constructed from Cr–Co milled from a disk using CAM.

The patients underwent a regimen of a strict home hygiene instruction, motivational support, and professional sessions. Dedicated soft brushes and ozone irrigation were recommended to keep the subtle virtual space between the mucosa and the rehabilitation structure clean.

### Follow-up and outcome measures

The patients were followed at 3-month intervals throughout the postsurgical period, as this was the standard practice among the clinics involved in the study. Based on both clinical and radiographic criteria described by Buser and colleagues [20], the implants were classified as successful or unsuccessful. The criteria for implant failure were as follows: (1) persistent patient complaints; (2) peri-implant suppurative infection; (3) fixture mobility; and (4) worsening radiolucency at the marginal bone level.

Marginal bone levels were assessed on periapical radiographs obtained using the long-cone paralleling technique with a loop film holder (Rinn, Dentsply Australia Pty, Ltd, Pacific Hwy, St. Leonards, NSW 2065, Australia). Radiographs were standardized by means of individual resin bites. The distance between the implant–abutment connection and the first bone-to-implant contact (fBIC) on mesial and distal surfaces was recorded. The scale was calibrated according to the width of the dental implant to achieve a unique pixel/mm ratio. The mean marginal bone level for each implant was computed by merging the mesial and distal variations. The marginal bone change was defined as the difference between the MBL at the last follow-up and the baseline MBL value, with negative values denoting a loss of bone height. All the implants were radiographically examined by one author who was unaware of the treatment procedure (EG) using an OsiriX DICOM viewer (Pixmeo SARL, 266 Rue de Bernex, CH-1233 Bernex, Switzerland).

The primary endpoint outcomes were as follows:

- Implant survival (the implant was present in the arch, supporting the prosthetic restoration)
- Implant success (not presenting the signs described above as indicative of failure)
- Prosthetic success (functional prosthetic restoration in the absence of any type of complication, either biological or mechanical)

The secondary outcomes were as follows:

- General patient satisfaction and quality of life (data were collected using a written questionnaire and the simplified Oral Health-Related Quality of Life (OHRQoL) questionnaire)
- Soft tissue stability (verified on digital photographs each time by the same operator, who was not involved in the surgery)

The PICO answer to the study was as follows: Does the flat one-bridge implant-prosthesis approach (I) affect or influence long-term implant and prosthetic success (O) in patients who require a full-arch fixed rehabilitation (P)?

### Statistical analysis

Statistical analysis was performed with R version 3.6.3 (2020-02-29), "Holding the Windsock" (www.r-project.org/), a free software environment for statistical computing and graphics. The incidence of implant failures and complications is expressed as absolute values and percentages (%). Cohort characteristics were analyzed as numbers (count or percentage), means (standard deviations [SD]), or medians (interquartile ranges [IQR]). To assess the effect of different variables on prosthesis success, Wilcoxon tests were used for scale variables, and chi-square tests were used for categorical variables. The time to failure was analyzed by Kaplan–Meier survival analysis. Implant survival was calculated at both the implant level and the patient level; with the patient as the statistical unit, the case was classified as a failure if even one implant failure occurred. Similarly, prosthetic success was calculated at both the restoration and patient levels. The effects of covariates on failure were analyzed by means of Mantel-Cox comparisons. The test used to compare immediate and nonimmediate implantation was the *nparLD* function within the *nparLD* package, which is highly reliable for nonparametric longitudinal data.

## Results

A total of 66 consecutive patients (30 males and 36 females, mean age 59.4 ± 10.1 years, ranging from 41 to 84 years) were included in the study, 9 of whom underwent bimaxillary treatment. In total, 75 arches were rehabilitated (45 maxillary and 30 mandibular). A total of 13 prostheses were supported by 4 implants, 2 prostheses were supported by 5 implants, 24 prostheses were supported by 6 implants, 9 prostheses were supported by 7 implants, 22 prostheses were supported by 8 implants, 1 prosthesis was supported by 9 implants, and 4 prostheses were supported by 10 implants. Table 1 shows the demographic characteristics of the study cohort.

**Table 1:** Demographics of the study population and relative chi-squared test for complication rate

Variable	N	Chi-squared test for complication rate
*Sex*		
Male	30	0.99
Female	36	
*Smoking habit*		
Smoker	24	0.56
Nonsmoker	48	
*Age group*		
< 60 years	37	0.98
≥ 60 years	35	
*Systemic condition*		
Antidepressant therapy	2	NA
Coronary heart disease	1	
Diabetes	1	
Hypertension	3	
Periodontal disease	17	
*Arch*		
Maxilla	45	0.71
Mandible	30	
*Oral hygiene*		
Good	43	0.42
Poor	29	

A total of 494 implants were placed, 367 of which were in postextraction sites (74.2%). Only 4 out of 494 implants were loaded in a delayed manner. All the other implants (490) were placed with an insertion torque of 35 Ncm or higher. No implant was excluded from the survival analysis.

**Fig. 7:**
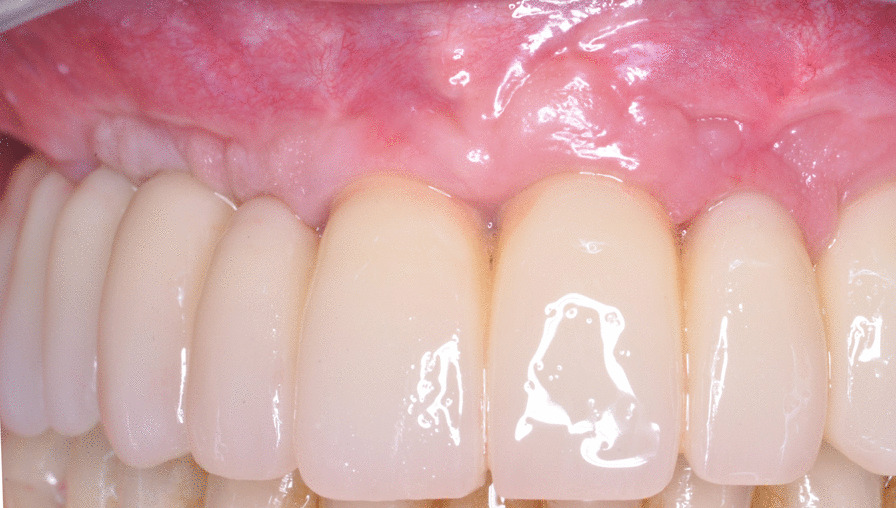
Intraoral photograph of the restoration at an 8-year follow-up visit

**Fig. 8:**
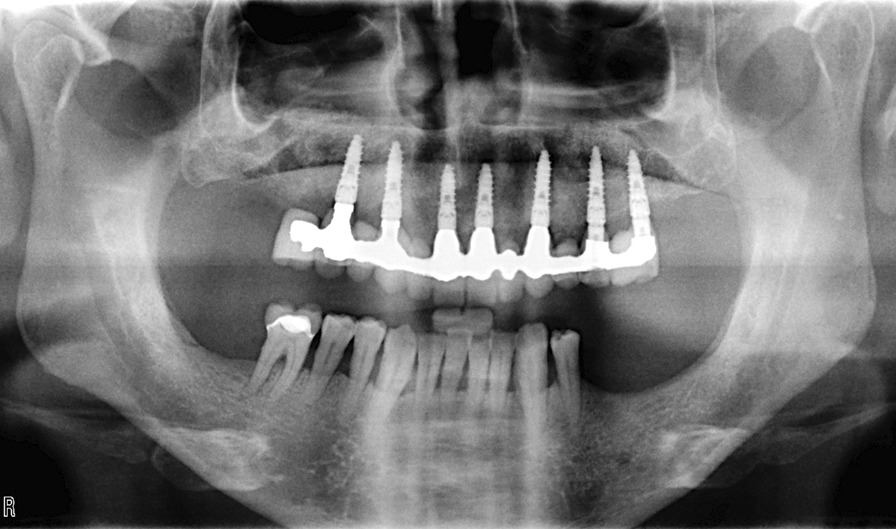
Radiological control at an 8-year follow-up visit

A flapless approach was used in 11 cases, and a regeneration procedure was used in 41 arches (almost 50% of the cohort). The soft tissue appeared healthy and mucositis-free at the time of provisional removal and definitive restoration delivery and at the last follow-up (Fig. 7). The radiological follow-up consistently confirmed substantial preservation of peri-implant marginal bone for almost the entire cohort of patients (Fig. 8). The mean marginal bone level was + 1.29 ± 0.90 mm at 6 months. Seven years after loading, the mean marginal bone level was -0.32 ± 0.50 mm.

The functional duration of the immediate prosthesis ranged from 82 to 168 months, and the median duration was 86 months. A total of 73 prostheses functioned for more than 7 years. A total of 56 patients still used their immediate prostheses at the end of our observation, despite having been warned that provisional prostheses are not intended for long-term use. The other patients opted to replace the immediate prosthesis with the final metal-acrylic prosthesis.

A total of 3 prostheses (4%) experienced complications during the entire functional period: in each case, the complication was related to the loss of one implant. However, the loss did not compromise the survival of the rehabilitation in any case. Thus, the cumulative success rate of the prostheses was 96% according to the Kaplan–Meier curve.

**Fig. 9:**
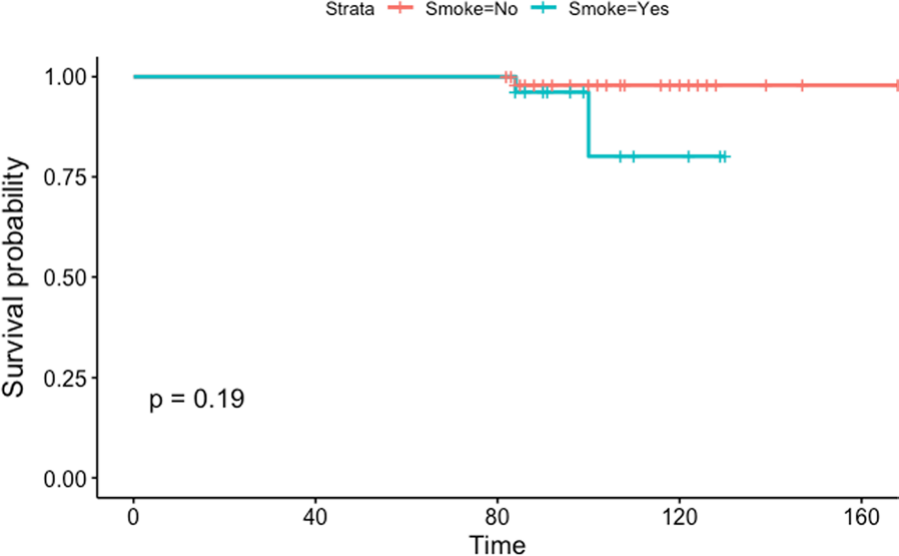
Graph of the Kaplan–Meier cumulative success rate for implants, stratified by smoking habit. The vertical axis shows the cumulative proportion of nonfailed implants

**Fig. 10:**
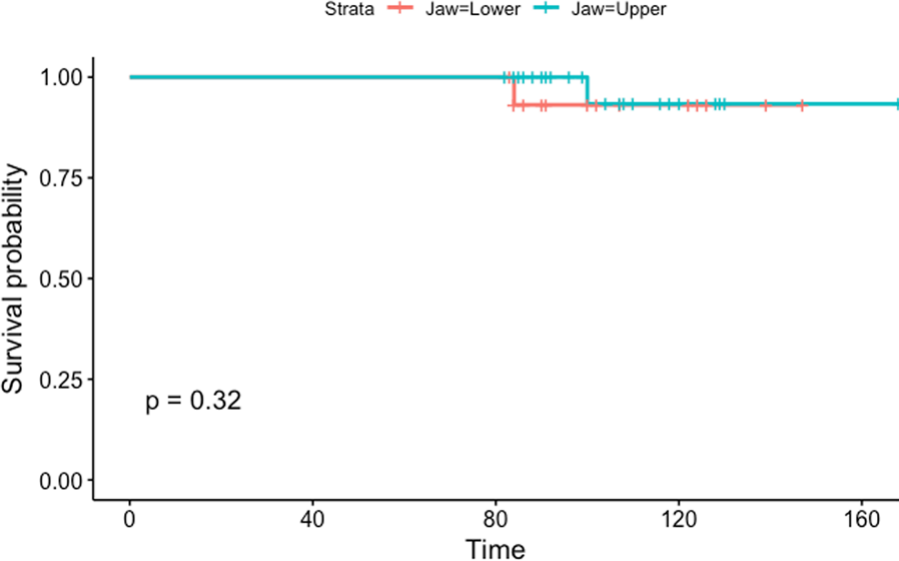
Graph of the Kaplan–Meier cumulative success rate for implants, stratified by jaw. The vertical axis shows the cumulative proportion of nonfailed implants

**Fig. 11:**
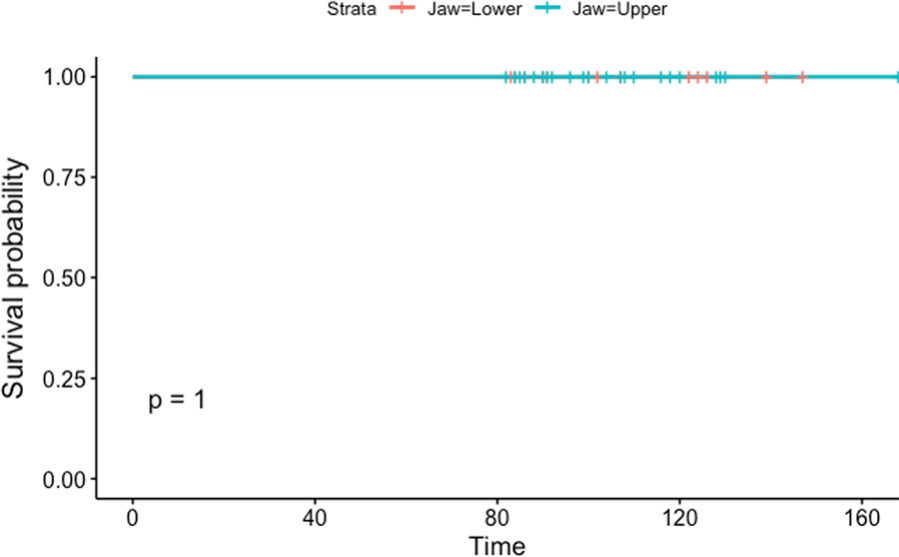
Graph of the Kaplan–Meier cumulative prosthesis survival, stratified by jaw. The vertical axis shows the cumulative proportion of nonfailed prostheses

The cumulative implant survival rate at 7 years was 99%. A total of 3 implants failed in three different patients, and one of the three failed implants was placed in a postextraction site. All of the implant failures occurred in patients who smoked; two were in the mandible, and one was in the upper jaw. Figure 9 shows the cumulative implant survival rate stratified by smoking habits, and Fig. 10 shows the cumulative implant survival rate stratified by arch. Figure 11 shows the cumulative prosthesis survival curve. The cumulative prosthesis survival rate was 100% at 86 months, which was the median for this study. However, all of the prostheses that exceeded 84 months of follow-up also survived.

With the exception of a few patients who required repeated occlusion adjustment and night-guard fabrication, no further mechanical complications occurred.

Twenty-seven subjects had anamnestic notes related to systemic conditions that did not prevent the intervention (16 patients reported a history of periodontal problems; 2 were taking antidepressants for minor depression; 5 had hypertension; 1 had cardiovascular problems; 1 was diabetic; 33 had poor oral hygiene at the beginning of the study, indicated by gingival bleeding and residual calculus). Twenty-five patients were considered smokers (more than 10 cigarettes per day).

The Cox proportional hazards regression model was used to evaluate the association between complications and several possible risk indicators at the patient level. The multivariable Cox proportional hazards regression model showed that the HR was always lower than 1 for all of the covariates. Thus, no significant influence could be determined for smoking habits, hygiene, systemic health, sex, age and so on.

The assessment of the patients' OHRQoL in relation to immediate-loading full-arch implant therapy indicated significant improvement in quality of life from the beginning of rehabilitation to the delivery of the final restoration. General patient satisfaction was high for the entire cohort immediately after surgery and at the last follow-up, and patient and clinician evaluations of the function and aesthetic acceptance of the implant rehabilitation were consistent.

## Discussion

This retrospective study suggested that the rehabilitation of edentulous patients or patients with failing dentition with immediate full-arch loading using the flat one-bridge technique is reliable and successful, even in cases of postextraction implant placement. The present approach focused on the passive fitting of the prosthetic framework over the surface of a flat abutment. The use of such abutments helps clinicians exploit the residual bone when there is a lack of volume at postextraction sites. Almost all of the rehabilitations successfully survived up to the 7-year follow-up visit; only 3 out of 75 prostheses presented complications, and those were related to a single implant failure: in each case, the survival of the prosthesis was still ensured.

Relevant literature describes success rates very similar to those of the present study [21, 22].

In 2018, Gallucci and colleagues reviewed the evidence regarding oral rehabilitations with different combinations of implant placement and loading protocols: immediate implant placement and loading, immediate implant placement with early loading, immediate implant placement with delayed loading, and late implant placement and late loading [23]. The analysis indicated that the highest survival and success scores occurred for the combination of immediate implant placement with early loading (98.2% and 98.7–100%, respectively). The authors suggested the importance of considering placement and loading time as a single denominator in the analysis of the overall success.

The few studies that assessed the outcome of full-arch restorations supported by postextractive implants and/or immediate and nonimmediate implants were not homogenous.

In the present study, most of the implants were placed in postextraction sites, and no differences in survival and success were noted between immediate implants and implants placed in healed ridges. This result is in line with previous literature on the same topic. Altintas and colleagues found that the success rate was the same for nonimmediate and immediate implants (97.8%), supporting the use of full-arch fixed prostheses [24]. More recently, Lerner and colleagues presented a retrospective clinical study of 110 implants (65 of which were postextraction) that showed a very high success rate for complete-arch fixed prostheses (98.2%) [25].

Most of the studies included a digital planned template to guide surgery when failing teeth were to be extracted and, thus, immediate implants were to be placed. This was not the case in the present study, in which implants were placed without the aid of a digitally manufactured surgical template. It must be highlighted that immediate implants might represent a challenge for ordinary surgeons, and it has been suggested that skillfulness might influence the clinical outcome of postextraction implants [9]. This is even more pronounced in the case of multi-implant positioning, which requires careful preoperatory diagnostic planning and intraoperative adaptability. In fact, primary stability is simply achieved wherever sufficient bone is available; therefore, longer implants should be used, as should the maximum number of implants.

The environment surrounding immediate postextraction implants is unique: from a biological perspective, it contains periodontal ligament remnants; from a mechanical perspective, peri-implant compression strain occurs during implant insertion; this phenomenon, coupled with heat transfer during drilling, creates a zone of apoptosis around the implant [26]. Osteogenesis in the alveolar fossae is primarily caused by blood clots around the implant; therefore, extensive blood lacunae around the implant surface represent the initial osteogenesis [27].

The positive success rate of immediate implants in the present study might be explained by the macroscopic mechanical passivation of the suprastructure that is mediated by the flat-to-flat connection.

Furthermore, the patented design of the Ossean implant surface has specific hydrophilic properties that enhance osteointegration in the first 4 weeks after surgery: in fact, the fractal, nanorough Ossean surface has been reported to influence cellular genetic expression or the fate of stem cells at the nano level, which in turn induces faster implant osteointegration [28].

The limitations of FoB are that the technique is not useful in cases of single edentulism. Furthermore, the technique depends on proper implant placement and the use of an appropriate surgical technique.

This was a retrospective study without a control group; therefore, its results may not be generalizable. The level of evidence of retrospective studies is inferior to that of prospective studies; furthermore, this study design is also prone to a posteriori misclassification biases. A further limitation of the study is the lack of data analysis regarding peri-implant mucosal inflammation. It must also be said that the entire approach could be further simplified with the introduction of a fully digital workflow [28].

The long-term use of implant-supported full-arch immediate prostheses usually leads to a high prevalence of prosthetic complications, and prosthesis fracture is the most common complication during the first year of loading. In the present study, no prosthetic fracture was reported, confirming that framework reinforcement (including metallic or even glass fiber reinforcement) could be the most important key to success during the osseointegration period. Glass fiber-reinforced acrylic immediate prostheses may function better in patients with removable dentures in the opposing jaw. It should be highlighted that there are numerous recent studies supporting the introduction of monolithic zirconia as a substitute for metal-acrylic prostheses, with favorable survival rates and low risk of fracture [29]. More comparative studies are needed to demonstrate these postulates.

Even if the flat abutment protocol can also be provided with a lower number of supporting implants, the optimal number of implants was always considered a minimum of 6 and 8 implants in the lower and upper arch, respectively. Only future studies will be helpful to ascertain the minimum number of implants to be required for prosthetic support with this type of protocol.

## Conclusion

In the case of FOB rehabilitations, this specific protocol may have some favorable ethical and social implications due to its good cost–benefit ratio and minimally invasive approach. It helps the clinician achieve a passive reciprocal relationship between implants and may also offer satisfying aesthetic results, good function and reduced trauma to the bone, thereby causing less reduction of the crest than other, more invasive protocols.

## Data Availability

All data are available.

## Abbreviations

IIP: Immediate implant placement
L-PRF: Leukocyte- and platelet rich fibrin
FOB: Flat one-bridge
